# Feasibility Study of IMNCI Guidelines on Effective Breastfeeding in a Rural Area of North India

**DOI:** 10.4103/0970-0218.42067

**Published:** 2008-07

**Authors:** Madhu Gupta, Arun Kumar Aggarwal

**Affiliations:** Department of Community Medicine, School of Public Health, Post Graduate Institute of Medical Education and Research, Chandigarh - 160012, India

## Introduction

Children younger than 3 years old commonly suffer from fever (27% prevalence in the previous 2-week period), acute respiratory infections (17%), diarrhoea (13%) and malnutrition (43%) - and often a combination of these conditions.(1) During the mid-1990s, the World Health Organization (WHO), in collaboration with UNICEF and many other agencies, institutions and individuals, responded to this challenge by developing a strategy known as Integrated Management of Childhood Illness (IMCI). Due to high neonatal mortality and morbidity in the country, the Government of India revised the strategy to be called Integrated Management of Neonatal and Childhood Illness (IMNCI). This strategy addresses various aspects of nutrition, immunization and other important elements of disease prevention and health promotion in addition to early detection and prompt management of cited childhood illnesses. Great emphasis was given on effective breast-feeding in IMNCI guidelines.(2) The present study was planned with the objective of implementing IMNCI guidelines of effective breastfeeding as an intervention and comparing the results in pre- and post-intervention period in rural areas.

## Materials and Methods

The study was conducted in a rural health and training centre covering the villages Kheri and Kakrali by School of Public Health, Department of Community Medicine, PGIMER, Chandigarh, India, from February to June 2005. Mothers with children aged < 2 years and those breastfeeding their babies at the time of study were included. The data collection instrument was a pre-tested proforma, which was designed using the standard IMNCI breastfeeding guidelines.(3) Data collection was done by a doctor (a postgraduate in community medicine), who was trained in IMNCI. The first part of the proforma dealt with general socio-demographic information about the mother including age, literacy level, caste, occupation, parity, etc. The second part of the proforma was used to record observations pertaining to the position of baby and its attachment with mother while breastfeeding. The awareness of the mother regarding breastfeeding time and interruptions during feeding was also assessed. Other responses like tiredness after breastfeeding, frequency of feeding, complaints in breasts were also noted.

After recording the pre-intervention observations related to position and attachment of the baby while breastfeeding, a demonstration of correct breastfeeding practices was made to mothers as per IMNCI guidelines. IMNCI chart booklet was used to illustrate correct and wrong breastfeeding practices. Post-intervention observations were done after a gap of 10-15 days using the same proforma. Data were analyzed using Epi Info 2000 statistical software. The paired *t* -test was used to test differences in observations between pre- and post-intervention periods. The differences were considered to be statistically significant at the 5% level.

## Results

Out of 32 mothers (14 from Kakrali village and 18 from Kheri village) with children aged < 24 months, 31 mothers were breastfeeding their babies and included in the study. The purpose of the first home visit was to record pre-intervention observations while breastfeeding. For recording post-intervention observations, we contacted 25 mothers in our second visit after a gap of 10 days, 5 mothers in our third visit after a gap of 15 days, and 2 mothers could not be contacted. The response rate was 93.5%. The socio-demographic profile of mothers shows that they were between 20 and 35 years; 42% were married for 1-3 years; 32% were primiparous; 22% were illiterate; 64% of mothers had infants of age < 6 months. All the mothers were homemakers.

Pre- and post-intervention observations related to position and attachment of the baby while breastfeeding are shown in Table 1. It was observed that following intervention, a significant number of mothers (82.8%) were keeping the baby close to them, and another significant observation (89.7%) was that the baby's neck was found to be straight or slightly bent backwards while breastfeeding. A significant number of babies were suckling deep and well in the post-intervention period. However, no significant change was observed in correction of these when all criteria were combined.

**Table 1:** Pre- and post-intervention observations pertaining to position and attachment in breastfeeding

Observations	Before intervention, *n* = 31	After intervention, *n* = 29	t, df = 28	Sig.(two-tailed)
Positioning				
Infant's body is turned towards the mother	16 (51.6)	25 (86.2)	4.1	0.03*
Infant's neck straight or slightly bent back	22 (71.0)	26 (89.7)	1.9	0*
Both arms supporting the baby	4 (12.9)	14 (48.3)	3.0	0.06
Infant's body close to mother	14 (45.2)	24 (82.8)	3.5	0.00*
Attachment				
Brings infant's upper lip to touch nipple	24 (77.4)	28 (96.6)	1.9	0.06
Waits for the infant to open mouth widely	27 (87)	29 (100)	1.7	0.08
Mouth wide open	16 (51.6)	25 (86.2)	2.7	0.01*
Chin touching breast	6 (19.4)	19 (65.5)	4.5	0*
Lower lip averted	22 (71)	24 (82.8)	1.1	0.26
Areola visible more above than below	17 (54.8)	24 (82.8)	2.8	0.01*
Child suckling deep and well	14 (45.2)	26 (89.7)	4.2	0*
Nose clear and not blocked	29(93.5)	28 (96.6)	0	1
Correct positioning	0	12(42)	4.5	0*
Correct attachment	3 (9.7)	12 (41.4)	3.8	0*
Both correct	0	3	1.8	0.08

* *P* < 0.05 Statistically Significant

Mothers had significantly shown improvement in breastfeeding time per session. As a post-intervention observation, it was noted that a significant number (21.4%) of mothers reported getting tired after breastfeeding. A reason given for this was holding the baby with both hands, and some mothers experienced back pain. It was observed that, with the increase in parity up to 3, the number of mothers adopting correct position and attachment increased in the post-intervention period. After a parity of >3, this number declined. As the age of baby may influence breastfeeding practice, out of 20 mothers with babies under 6 months, 8 mothers (40%) had shown correct position following intervention as compared to 27% (3/11) mothers with babies over 6 months. Similarly, with respect to attachment, 50% (10) of mothers with babies under 6 months had shown improvement following intervention as compared to 18% (2/11) mothers with babies over 6 months. However, the association observed between parity, age of baby, literacy of mothers and effective breastfeeding was not significant statistically.

## Discussion

Many studies have documented the usefulness of mother's milk, but literature is scanty regarding the prevalence of breastfeeding practices.(4) To our knowledge, there was no study to document the cultural acceptability of breastfeeding techniques to rural Indian women. This study is the first of its kind in this region. There are reports available on the overall impact on infant mortality rate as a result of implementation of IMNCI strategy in many developing countries.(5) However, the impact of IMNCI guidelines on effective breastfeeding has not been studied. In this study, the IMNCI guidelines of breastfeeding helped mothers in bringing the babies close to them and turned towards them. By adopting these two techniques, the attachment of the baby, especially suckling, significantly improved. Since the baby was suckling deep and well, it could suck for long, as was evident from increased feeding time after the intervention. However, no significant improvement was observed in supporting the baby with both hands because of the menace of house flies; as a result supporting the baby with both hands was impractical as one hand was needed to drive the flies away. North Indian women usually wear the *salwaar kameej.* To feed the baby, the mother had to lift her *kameej* to her chest and hold it with her one hand. Moreover, back pain as a result of supporting the baby with both hands prevented some mothers from using this particular technique while breastfeeding. In such situations, recommending breastfeeding as per the IMNCI guidelines becomes difficult. It is concluded that although IMNCI guidelines of breastfeeding are a useful tool for effective breastfeeding in rural areas, socio-cultural barriers should be taken care of while designing these guidelines, which are to be implemented in developing countries like India.
